# Supplementary material for: The Small RNA Universe of Capitella teleta
Source: Front Mol Biosci. 2022 Feb 25;9:802814. doi: 10.3389/fmolb.2022.802814 (PMC8915122; doi:10.3389/fmolb.2022.802814)
Supplement: Supplementary file 1 [file DataSheet1.ZIP › Supplement/known/CAPTEscaffold_394_20716.pdf]

Provisional ID	:	CAPTEscaffold_394_20716
Score total	:	12820.1
Score for star read(s)	:	3.9
Score for read counts	:	12810.5
Score for mfe	:	1.1
Score for randfold	:	1.6
Score for cons. seed	:	3
Total read count	:	25139
Mature read count	:	25119
Loop read count	:	0
Star read count	:	20

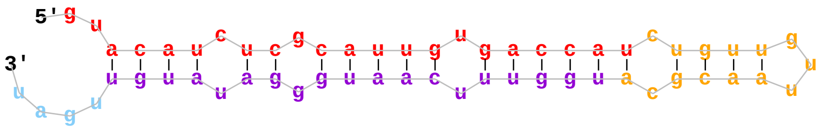

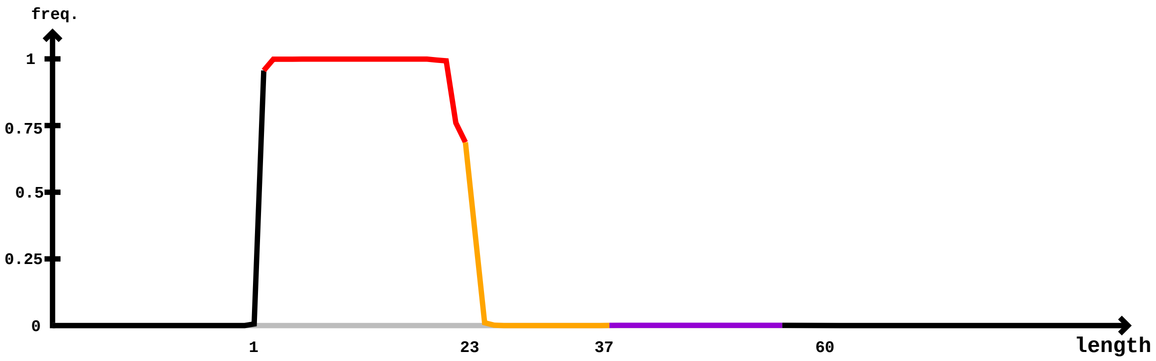

Mature **Star**

```
5'- gaagcagggcgaaguuuucuguacaucucgcauugugaccaucuguuguuaacgcaugguuucaaugggauauguugauaaagcuuccgucgaggucaacauccgaggcu  -3'   obs
    gaagcagggcgaaguuuucuguacaucucgcauugugaccaucuguuguuaacgcaugguuucaaugggauauguugauaaagcuuccgucgaggucaacauccgaggcu         exp
    gaagcagggcgaaguuuucuguacaucucgcauugugaccaucuguuguuaacgcaugguuucaaugggauauguugauaaagcuuccgucgaggucaacauccgaggcu         known
    ..........((((((((....((((.((.(((((.((((((.((((...)))).)))))).))))).)).))))....)))))))).(((..((.......))..))).   reads   mm   sample

    ...................uguacaucucgcauuguga........................................................................   1   0   seq
    ...................uguacaucucgcauugugG........................................................................   1   1   seq
    ...................uguacaucucgcauugugacc......................................................................   63  0   seq
    ...................Aguacaucucgcauugugacc......................................................................   1   1   seq
    ...................uguacaucucgcauugugacca.....................................................................   2   0   seq
    ...................uguacaucucgcauugugaccau....................................................................   81  0   seq
    ...................uguacaucucgcauugugaccauU...................................................................   3   1   seq
    ....................guacaucucgcauugugG........................................................................   13  1   seq
    ....................guacaucucgcauuguga........................................................................   75  0   seq
    ....................guacaucucgcauugAga........................................................................   1   1   seq
    ....................gAacaucucgcauugugac.......................................................................   1   1   seq
    ....................guacaucucgcauugugaA.......................................................................   1   1   seq
    ....................Uuacaucucgcauugugac.......................................................................   2   1   seq
    ....................guacaucucgcauugugac.......................................................................   67  0   seq
    ....................guacaucucgcauugugaU.......................................................................   1   1   seq
    ....................guacGucucgcauugugacc......................................................................   1   1   seq
    ....................guacaucucUcauugugacc......................................................................   1   1   seq
    ....................Cuacaucucgcauugugacc......................................................................   5   1   seq
    ....................gAacaucucgcauugugacc......................................................................   6   1   seq
    ....................guacaucucgcauugugUcc......................................................................   4   1   seq
    ....................guacaucuGgcauugugacc......................................................................   1   1   seq
    ....................guacaucAcgcauugugacc......................................................................   1   1   seq
    ....................guGcaucucgcauugugacc......................................................................   3   1   seq
    ....................Nuacaucucgcauugugacc......................................................................   1   1   seq
    ....................Auacaucucgcauugugacc......................................................................   9   1   seq
    ....................guacaucuUgcauugugacc......................................................................   3   1   seq
    ....................guacaucucgcauugugCcc......................................................................   3   1   seq
    ....................guacaucucgcaAugugacc......................................................................   3   1   seq
    ....................Uuacaucucgcauugugacc......................................................................   13  1   seq
    ....................guacaucucgcauugAgacc......................................................................   3   1   seq
    ....................guacaucucgAauugugacc......................................................................   1   1   seq
```

```
                    Mature                              Star
gaagcagggcgaaguuuucuguacaucucgcauugugaccaucuguuguuaacgcaugguuucaaugggauauguugauaaagcuuccgucgaggucaacauccgaggcu
....................guacaucucgcauCgugacc......................................................................   1       1    seq
....................guacauUucgcauugugacc......................................................................   1       1    seq
....................guacaucucgcauugugGcc......................................................................   2       1    seq
....................guacaGcucgcauugugacc......................................................................   1       1    seq
....................guacaucucgcauugugacc......................................................................   5485    0    seq
....................guacaAcucgcauugugacc......................................................................   1       1    seq
....................guacaucucgcauuguAacc......................................................................   3       1    seq
....................guacaucucgcauugugacU......................................................................   53      1    seq
....................guacaucucgcauuCugacc......................................................................   1       1    seq
....................guacaucucgcauugugacA......................................................................   2       1    seq
....................guacaucucCcauugugacc......................................................................   1       1    seq
....................guacaucucgcauugugaAc......................................................................   2       1    seq
....................guacUucucgcauugugacc......................................................................   1       1    seq
....................guacaucucgcauAgugacca.....................................................................   1       1    seq
....................guacaucucgcauuguAacca.....................................................................   1       1    seq
....................guacaucucgcauugugacca.....................................................................   1772    0    seq
....................guacaucucgcauugugaccU.....................................................................   5       1    seq
....................guacaucucgAauugugacca.....................................................................   1       1    seq
....................Uuacaucucgcauugugacca.....................................................................   2       1    seq
....................guacaucucgcauugugGcca.....................................................................   1       1    seq
....................guacaucucgcauugugaAca.....................................................................   1       1    seq
....................gAacaucucgcauugugacca.....................................................................   3       1    seq
....................guacaAcucgcauugugacca.....................................................................   1       1    seq
....................guacaucucgcauugCgacca.....................................................................   1       1    seq
....................guacaucuUgcauugugacca.....................................................................   1       1    seq
....................guacaucucgcauugugaccG.....................................................................   1       1    seq
....................Cuacaucucgcauugugacca.....................................................................   3       1    seq
....................guacaucucgcauugAgacca.....................................................................   1       1    seq
....................guacaucucgcauugugacAa.....................................................................   2       1    seq
....................guacaucuAgcauugugacca.....................................................................   1       1    seq
....................guaAaucucgcauugugacca.....................................................................   1       1    seq
....................guacaucucgcauCgugacca.....................................................................   22      1    seq
....................guacaucucgcauuUugaccau....................................................................   1       1    seq
....................Nuacaucucgcauugugaccau....................................................................   2       1    seq
....................guacUucucgcauugugaccau....................................................................   1       1    seq
....................guacaucAcgcauugugaccau....................................................................   8       1    seq
....................guacaucucgcauugugUccau....................................................................   3       1    seq
....................guUcaucucgcauugugaccau....................................................................   2       1    seq
....................gAacaucucgcauugugaccau....................................................................   14      1    seq
....................guacaucucgcauuAugaccau....................................................................   1       1    seq
....................guCcaucucgcauugugaccau....................................................................   1       1    seq
....................guacaucucgcauugugaccaA....................................................................   3       1    seq
....................guacaucucgcauuguCaccau....................................................................   1       1    seq
....................guacaucucgcauugAgaccau....................................................................   12      1    seq
....................guacaucucgcauuguAaccau....................................................................   7       1    seq
....................guacGucucgcauugugaccau....................................................................   5       1    seq
....................guacaucucgcauugugacAau....................................................................   15      1    seq
....................guacaucucgcaCugugaccau....................................................................   1       1    seq
....................guacaucuUgcauugugaccau....................................................................   11      1    seq
....................guacaucucgcauugugaccaC....................................................................   1       1    seq
....................guacaucucAcauugugaccau....................................................................   3       1    seq
....................guacaucucgcauugugacUau....................................................................   6       1    seq
....................guacaucucgcauugugGccau....................................................................   2       1    seq
....................guacaucuAgcauugugaccau....................................................................   1       1    seq
....................guacaucucgcauugugaccGu....................................................................   5       1    seq
....................guacaucucgcauAgugaccau....................................................................   2       1    seq
....................guacaAcucgcauugugaccau....................................................................   6       1    seq
....................guacauUucgcauugugaccau....................................................................   1       1    seq
....................guGcaucucgcauugugaccau....................................................................   19      1    seq
....................guacaucucgcauuguUaccau....................................................................   2       1    seq
....................guacaucucgcauugugCccau....................................................................   3       1    seq
....................guacaucCcgcauugugaccau....................................................................   1       1    seq
....................guacaucucgcauugCgaccau....................................................................   1       1    seq
....................guacaucucgcauugugaccau....................................................................   13622   0    seq
....................gCacaucucgcauugugaccau....................................................................   1       1    seq
....................gGacaucucgcauugugaccau....................................................................   3       1    seq
....................guaUaucucgcauugugaccau....................................................................   1       1    seq
....................guacauAucgcauugugaccau....................................................................   6       1    seq
....................guacaucucgcauuCugaccau....................................................................   1       1    seq
....................guacaucucgcaAugugaccau....................................................................   6       1    seq
```

Mature Star

```
gaagcagggcgaaguuuucuguacaucucgcauugugaccaucuguuguuaacgcaugguuucaaugggauauguugauaaagcuuccgucgaggucaacauccgaggcu
....................guacaucucgcGuugugaccau....................................................................  3     1  seq
....................guacaucucgcauugugaccaG....................................................................  7     1  seq
....................guacaucucUcauugugaccau....................................................................  3     1  seq
....................guacaucucgAauugugaccau....................................................................  1     1  seq
....................guaAaucucgcauugugaccau....................................................................  2     1  seq
....................guacaucuGgcauugugaccau....................................................................  1     1  seq
....................Cuacaucucgcauugugaccau....................................................................  8     1  seq
....................Uuacaucucgcauugugaccau....................................................................  19    1  seq
....................guacaucucgGauugugaccau....................................................................  1     1  seq
....................guacaucucgcauCgugaccau....................................................................  1     1  seq
....................Auacaucucgcauugugaccau....................................................................  4     1  seq
....................guacaucucgcauugugaccauc...................................................................  36    0  seq
....................guacaucucgcauugugaccauA...................................................................  308   1  seq
....................guacaucucgcauugugaccauU...................................................................  2000  1  seq
....................guacaucucgcauugugaccauG...................................................................  2     1  seq
....................guacaucucgcauugugaccaucu..................................................................  1     0  seq
....................guacaucucgcauugugaccauAu..................................................................  16    1  seq
....................guacaucucgcauugugaccauUu..................................................................  69    1  seq
....................guacaucucgcauugugaccaucug.................................................................  35    0  seq
....................guacaucucgcauugugaccaucGg.................................................................  1     1  seq
.....................Nacaucucgcauugugacc......................................................................  1     1  seq
.....................Aacaucucgcauugugacc......................................................................  1     1  seq
.....................uacGucucgcauugugacc......................................................................  1     1  seq
.....................uacaucucgcauugugacc......................................................................  172   0  seq
.....................uacaucucgcauugugacca.....................................................................  10    0  seq
.....................uacaucuUgcauugugaccau....................................................................  1     1  seq
.....................Gacaucucgcauugugaccau....................................................................  1     1  seq
.....................uacaucucgcauugugaccau....................................................................  320   0  seq
.....................uacaucucgcauugugaccaA....................................................................  1     1  seq
.....................uGcaucucgcauugugaccau....................................................................  1     1  seq
.....................uacaucucgUauugugaccau....................................................................  1     1  seq
.....................uaGaucucgcauugugaccau....................................................................  1     1  seq
.....................uacaucucgcauugugaccauA...................................................................  2     1  seq
.....................uacaucucgcauugugaccauc...................................................................  352   0  seq
.....................uacaucucgcauugugaccauU...................................................................  51    1  seq
.....................Aacaucucgcauugugaccauc...................................................................  4     1  seq
.....................uacaucucgcauugAgaccauc...................................................................  1     1  seq
.....................uaGaucucgcauugugaccauc...................................................................  1     1  seq
.....................uacaCcucgcauugugaccaucu..................................................................  1     1  seq
.....................uacaucucgcauugugaccaucu..................................................................  138   0  seq
.....................uacaucucgcauugugaccaucG..................................................................  1     1  seq
.....................Aacaucucgcauugugaccaucu..................................................................  1     1  seq
........................aucucgcauugugaccauU...................................................................  11    1  seq
.......................................................ugguuucaaugggauaugu...................................  13    0  seq
.......................................................ugguuucaaugggauauguuga................................  3     0  seq
........................................................gguuucaaugggauauguuga................................  1     0  seq
........................................................gguuucaaugggauauguugau...............................  2     0  seq
........................................................gguuucaaugggauauguugauU..............................  1     1  seq
```
